# Malnutrition and infection lead to poor prognosis and heavy financial burden of patients with chronic heart failure

**DOI:** 10.3389/fcvm.2022.1045262

**Published:** 2022-12-01

**Authors:** Xu Jia, Xing-long Yu, Bin Lu, Yuan-yuan Shang, Long-fei Shen, Yu-lin Li, Wei Zhang, Ming Zhong, Lu Han, Zhi-Hao Wang

**Affiliations:** ^1^The Key Laboratory of Cardiovascular Remodeling and Function Research, Chinese Ministry of Education, Chinese National Health Commission and Chinese Academy of Medical Sciences, The State and Shandong Province Joint Key Laboratory of Translational Cardiovascular Medicine, Department of Cardiology, Cheeloo College of Medicine, Qilu Hospital, Shandong University, Jinan, Shandong, China; ^2^Department of Cardiology, People's Hospital of Lixia District of Jinan, Jinan, Shandong, China; ^3^Department of General Practice, Qilu Hospital of Shandong University, Jinan, China; ^4^Department of Geriatric Medicine, Shandong Key Laboratory of Cardiovascular Proteomics, Qilu Hospital, Cheeloo College of Medicine, Shandong University, Jinan, China

**Keywords:** chronic heart failure, malnutrition, infection, CONUT score, prognosis, financial burden

## Abstract

**Background:**

Chronic heart failure (CHF) is a major public health concern, as it is associated with poor prognosis and heavy financial burden. In recent years, there has been increasing interest in medications for CHF in China, but few studies pay attention to the effects of nutrition and infection.

**Methods and results:**

This was a retrospective study collected patients with CHF admitted to the Department of Cardiology of Qilu Hospital of Shandong University from January 2017 to May 2018. Patients were classified according to the prognosis and the financial burden. Through comparison and regression analysis, we found that the factor associated with worse prognosis were decreased heart rate, albumin and prealbumin; β-blockers and mineralocorticoid receptor antagonism (MRA) were the factor improved the prognosis of patients with CHF; the factor overburdening financial condition were infection, decreased prealbumin, high Alanine aminotransferase (ALT), usage of recombinant human brain natriuretic peptide (rhBNP) and Levosimendan; aspirin and Sacubitril/Valsartan were the factor releasing financial burden of patients with CHF. Then, we grouped by Controlling Nutritional Status (CONUT) score, which enabled evaluation of the patient's protein reserve and immune defenses. Patients in the malnutrition group had higher infection ratios, longer hospital stays, and greater hospital expenses than the normal group. The improvement ratios of therapeutic outcomes in the moderate or severe malnutrition group were lower than in the normal and mild malnutrition group.

**Conclusion:**

Malnutrition and infection caused poor prognosis and increased financial burden of patients with CHF. The high CONUT score indicated the CHF patient's unfavorable prognosis and heavy financial burden.

## Introduction

Heart failure (HF) is a chronic, serious, spontaneously progressive cardiovascular disease which is the end stage of many heart diseases, including ischemic heart disease, hypertension, dilated cardiomyopathy, and heart valve disease. It was clear that HF had been growing public health problem in China as a result of aging of the population and improving survival of patients with coronary artery disease (1). From the “Treatment recommendations for chronic systolic heart failure” (2) published in 2002 to the “Guidelines for the diagnosis and treatment of heart failure in China 2018” (3) released in 2018, China has successively released and updated multiple versions of HF guidelines and consensus. However, the one-year mortality ratio of patients with HF in China increased from 6.2% (4) in 2000 to 7% (5) in 2014. At the same time, the per capita hospital expenses of patients with HF gradually increased from 5,736.0 Yuan (6) in 2003 to 8,423.8 Yuan in 2016 (7).

Pharmacotherapy is recommended as the first line therapy on CHF (8, 9). But in recent years, some researchers have focused on factors other than pharmacotherapy, for instance nutrition and infection (10–12). For patients with CHF, Gastrointestinal edema caused by fluid retention often leads to in appetence, and energy intake reduction over a long period of time leads to malnutrition. As a source of power for blood circulation, the pump function of the heart is severely affected (13). Further aggravate the edema and form a vicious circle. Similarly, malnutrition leads to hypoimmunity and is more vulnerable to infection (14). Infection is the majority reason for patients with CHF to readmission, (15) which ultimately increases the economic burden of patients. At present, few study pay attention to the relationship between nutrition, infection and CHF in China. Hence, the aim of this study was to identify whether nutrition and infection affected the prognosis and financial burden of patients with CHF in China.

Controlling Nutritional Status (CONUT) is a scoring system that is used to assess the host's nutrition and immune states (16). CONUT is calculated from the serum albumin concentration, total blood cholesterol level, and total peripheral lymphocyte count. Some studies have reported the usefulness of CONUT for predicted survival ratio of cancer patients (17, 18), but there have been few reports for patients with CHF in China.

## Methods

A total of 500 cases with CHF which were admitted to the Department of Cardiology of Qilu Hospital of Shandong University from January 2017 to May 2018 were collected in our study. The selection criteria were established: (1) There were symptoms and signs of typical HF, such as dyspnea, fatigue, fluid retention, wet rales in the lungs, etc; (2) At least one auxiliary examination reflecting cardiac structure and/or function supported the diagnosis of HF, such as cardiac ultrasound or N-terminal-pro-B-type natriuretic peptide (NT-proBNP) ≥125 pg/mL; (3) Duration of disease ≥3 months. Exclusion criteria: (1) Age <18 years. (2) Automatic discharge or death within 24 h of admission.

We determined the patient's therapeutic outcomes by comparing the patient's condition, cardiac ultrasound, and NT-proBNP levels on admission with on discharge. Death, transfer to ICU or against advise discharge due to deterioration of heart condition, cardiac ultrasonic ejection fraction decreases or NT-proBNP increase before discharge, the above conditions were divided into deterioration group. Contrary to the above conditions were divided into improvement group. According to CONUT score, patients are divided into normal group (0–1), mild malnutrition group (2–4), moderate or severe malnutrition group (>5) (Supplementary Table S1). The patient's socio-demographic characteristics, medical history, discharge diagnosis, hospital expenses, hospital stays, laboratory results, and medication were collected through the inpatient case system. Data were sorted according to clinical data standards for HF recommended by the American College of Cardiology and American Heart Association (19).

SPSS 20.0 statistical software was used for analysis. If data conforming to the normal distribution, mean and standard deviation (SD) were used, and comparison between the two groups was performed by independent sample *t*-test. If data were skewed, median and interquartile range (IQR) were used, and the comparison between the two groups used the rank sum test. Counting data were described as frequency and percentage, and chi-square test was used for comparison between the two groups. Risk factors were analyzed by non-conditional logistic regression analysis, and influencing factors of re-admission interval were analyzed by linear regression. All tests were two-sided, and the differences were statistically significant at *p* < 0.05.

## Results

A total of 500 hospitalized cases with CHF were included in our study. Patients' mean age was 64.8 ± 14.8 years. Among the etiologies of HF, ischemic heart disease accounted for the highest proportion, followed by hypertension. Among the inducements of HF, infection accounted for the highest proportion. Among the auxiliary examinations to assess the condition of patients with CHF, the use of ECG accounted for the highest proportion. During and after hospitalization, diuretics were used most frequently, followed by mineralocorticoid receptor antagonism (MRA), β-blockers, angiotensin converting enzyme inhibitor (ACEI), and angiotensin II receptor blocker (ARB) (Supplementary Table S2).

## Prognosis

The readmission interval represents the frequency of patient's deterioration in a period of time. Our study used therapeutic outcomes and readmission interval to assess the long term prognosis of patients with CHF.

Compared with the improvement group, the proportion of males, prealbumin, albumin, lymphocyte, red blood cells and hemoglobin in the worsening group were lower (*p* < 0.05), and neutrophil and NT-proBNP were higher (*p* < 0.05). The usage ratios of ACEI/ARB, β-blockers, and levosimendan were lower in the worsening group (*p* < 0.05), and cedilanid was higher (Table 1).

**Table 1:** Factors influencing the therapeutic outcomes in patients with CHF.

	**Improvement group(*N =* 480)**	**Worsening group(*N =* 20)**	***p***
**Basic information**			
Male sex, *n* (%)	275(57.3%)	7(35.0%)^*^	0.049
Age, years, mean(SD)	64.6 ± 14.7	67.5 ± 18.0	0.398
SBP, mmHg, mean(SD)	126.0 ± 22.4	117.8 ± 27.4	0.109
DBP, mmHg, mean(SD)	74.5 ± 14.9	70.2 ± 16.6	0.212
HR, bpm, mean(SD)	81.9 ± 19.8	87.6 ± 25.6	0.215
HT History, *n* (%)	241(50.2%)	6(30.0%)	0.077
DM History, *n* (%)	135(28.1%)	6(30.0%)	0.855
CAD History, *n* (%)	184(38.3%)	9(45.0%)	0.548
Infection, *n* (%)	70(14.6%)	5(25.0%)	0.338
**Laboratory results**			
ALT, U/L, IQR	19.0(12.0, 30.0)	18.5(14.5, 78.3)	0.441
AST, U/L, IQR	23.0(18.0, 31.0)	24.0(21.0, 72.5)	0.205
TBIL, umol/L, IQR	15.4(10.7, 24.0)	21.4(9.4, 42.2)	0.307
AKP, U/L, mean(SD)	80.2 ± 30.6	87.8 ± 39.3	0.282
GGT, U/L, IQR	40.0(25.0, 75.5)	52.0(30.5, 105.8)	0.214
TC, mmol/L, mean(SD)	4.0 ± 1.1	3.6 ± 1.0	0.140
TG, mmol/L, mean(SD)	1.3 ± 1.0	1.1 ± 0.6	0.360
FBG, mmol/L, mean(SD)	5.5 ± 1.9	5.9 ± 3.3	0.624
Cr, umol/L, mean(SD)	95.6 ± 47.9	114.3 ± 87.2	0.365
BUN, mmol/L, IQR	6.5(5.1, 8.9)	6.8(5.0, 11.0)	0.395
PA, mg/dL, mean(SD)	19.0 ± 6.5	14.1 ± 5.9*	0.001
ALB, g/L, mean(SD)	39.5 ± 4.8	36.6 ± 5.3*	0.007
LDH, U/L, mean(SD)	271.9 ± 130.9	324.7 ± 155.0	0.127
UA, umol/L, mean(SD)	433.9 ± 151.7	466.9 ± 170.7	0.441
WBC, × 10^9^, mean(SD)	6.8 ± 2.1	6.8 ± 2.3	0.869
NEU, %, mean(SD)	65.4 ± 10.0	71.0 ± 10.2*	0.019
LYM, × 10^9^, mean(SD)	1.6 ± 0.6	1.2 ± 0.5*	0.007
RBC, × 10^12^, mean(SD)	4.4 ± 0.8	4.1 ± 0.7*	0.047
PLT, × 10^9^, mean(SD)	215.8 ± 84.8	184.2 ± 75.4	0.110
HGB, g/L, mean(SD)	132.8 ± 24.4	120.1 ± 20.5*	0.026
NT-proBNP, pg/mL, IQR	3800.0(1787.0, 7554.0)	7922.0(3395.8, 12950.0)*	0.015
**Medication**			
ACEI/ARB, *n* (%)	373(77.7%)	10(50.0%)*	0.009
β-blockers, *n* (%)	407(84.8%)	9(45.0%)*	<0.001
MRA, *n* (%)	420(87.5%)	17(85.0%)	1.000
Diuretics, *n* (%)	451(94.0%)	20(100.0%)	0.519
Sacubitril/Valsartan, *n* (%)	33(6.9%)	1(5.0%)	1.000
Ivabradine, *n* (%)	39(8.1%)	2(10.0%)	1.000
Aspirin, *n* (%)	301(62.7%)	11(55.0%)	0.486
Statin, *n* (%)	300(62.5%)	9(45.0%)	0.115
Digoxin, *n* (%)	182(37.9%)	6(30.0%)	0.474
Cedilanid, *n* (%)	251(52.3%)	15(75.0%)*	0.046
Levosimendan, *n* (%)	178(37.1%)	3(15.0%)*	0.044
rhBNP, *n* (%)	112(23.3%)	8(40.0%)	0.149

* *p*-value < 0.05. SBP, Systolic pressure; DBP, Diastolic blood pressure; HR, Heart rate; HT, Hypertension; DM, Diabetes; CAD, Coronary heart disease; ECG, Electrocardiogram; ALT, Alanine aminotransferase; AST, Aspartate aminotransferase; TBIL, Total bilirubin; AKP, Alkaline phosphatase; GGT, Glutamyl transpeptidase; TC, Total cholesterol; TG, Triglyceride; Cr, Creatinine; BUN, Blood urea nitrogen; PA, Prealbumin; ALB, Albumin; LDH, Lactate dehydrogenase; UA, Uric acid; WBC, Leukocyte; NEU, Neutrophil; LYM, Lymphocyte; RBC, Red blood cells; PLT, Platelets; HGB, Hemoglobin; NT-proBNP, N-terminal-pro-B-type natriuretic peptide; ACEI, Angiotensin converting enzyme inhibitor; ARB, Angiotensin II receptor antagonist; MRA, Mineralocorticoid receptor antagonism; rhBNP, Recombinant human brain natriuretic peptide.

Logistic regression was performed using the therapeutic outcomes of patients with CHF as the dependent variable, and basic information, laboratory results, medicine as independent variables. The results suggested that low albumin was a risk factor that worse therapeutic outcomes in patients with CHF, and β-blockers were protective factors that improved therapeutic outcomes of patients with CHF (Table 2).

**Table 2:** Logistic regression analysis of therapeutic outcomes in patients with CHF.

	**β**	**OR**	**95%CI**	***p***
ALB(g/L)	−0.059	0.943	0.922~0.964	<0.001
β-blockers	−1.560	0.210	0.068~0.651	0.007

ALB, Albumin.

**Figure 1:**
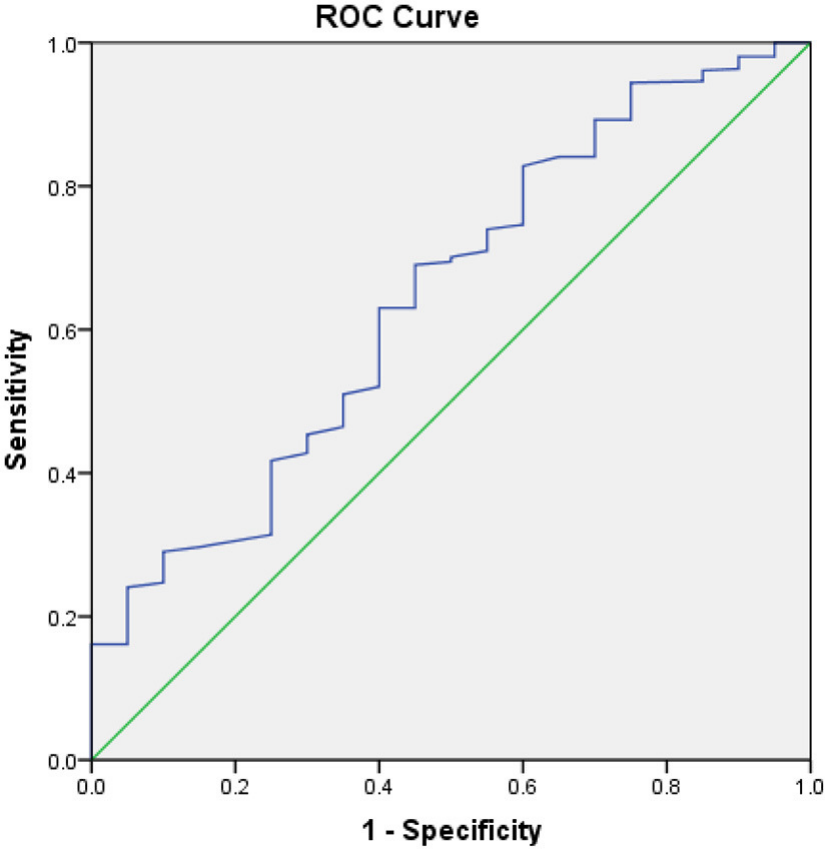
ROC analysis of outcomes of patients with CHF. The area under the ROC curve is 0.644, Sensitivity is 0.690, Specificity is 0.550, 95% CI [0.520–0.767], *p* = 0.029.

ROC curve was used to evaluate the value of albumin on the therapeutic outcomes of patients with CHF, which indicated 37.5g/L was the optimal cut-off point for albumin to predict the therapeutic outcomes of patient with CHF. The AUC of CONUT was 0.644 (95%CI 0.520–0.767). The sensitivity was 0.690 and the specificity was 0.550, respectively (Figure 1).

The median readmission interval for patients with CHF was 68.5 days. Linear regression analysis was performed with the readmissions interval as the dependent variable, and basic information, laboratory results, medicine as independent variables. The results showed that: heart rate, prealbumin, and usage of MRA were positively correlated with the hospital interval of patients with CHF (Table 3).

**Table 3:** Linear regression analysis of readmission interval for patients with CHF.

	**Unstandardized Coefficients β**	**Standardized Coefficients β**	***p***
**Basic information**			
HR	1.443	0.711	<0.001
**Laboratory results**			
PA(mg/L)	6.264	0.713	<0.001
**Medicine**			
MRA	132.061	0.689	<0.001

HR, Heart rate; PA, Prealbumin; MRA, Mineralocorticoid receptor antagonism.

## Financial burden

Patients with CHF needed professional caregiver or family member to take care of them. Hospital stays represent the cost of professional caregiver or family member. Therefore, in addition to hospital expenses, hospital stays were also used to assess the financial burden of patients with CHF.

The median of hospital stays for patients with CHF was 11 days. We divided all patients into the short hospital stays group and the long hospital stays group based on the median of hospital stays. Compared with the short hospital stays group, patients in the long hospital stays group with infection showed a higher proportion (*p* < 0.05). The levels of Aspartate aminotransferase (AST), alkaline phosphatase (AKP), lactate dehydrogenase (LDH), neutrophil, NT-proBNP were higher (*p* < 0.05), and albumin, hemoglobin were lower (*p* < 0.05). In terms of medicine, the ratios of diuretics, recombinant human brain natriuretic peptide (rhBNP) were higher in the long hospital stays group than in the short hospital stays group (*p* < 0.05). The ratios of aspirin was lower in the long hospital stays group (*p* < 0.05) (Table 4).

**Table 4:** Factors influencing the hospital stays of patients with CHF.

	**Short hospital stays group (*N* = 248)**	**Long hospital stays group (*N* = 252)**	***p***
**Basic information**			
Male sex, *n* (%)	146(58.9%)	136(54.0%)	0.269
Age, years, mean(SD)	65.0 ± 15.0	64.6 ± 14.7	0.761
SBP, mmHg, mean(SD)	124.6 ± 20.9	126.8 ± 24.3	0.284
DBP, mmHg, mean(SD)	74.2 ± 15.2	74.4 ± 14.8	0.835
HR, bpm, mean(SD)	81.7 ± 18.7	82.4 ± 21.5	0.696
HT History, *n* (%)	120(48.4%)	127(50.4%)	0.653
DM History, *n* (%)	70(28.2%)	71(28.2%)	0.990
CAD History, *n* (%)	99(39.9%)	94(37.3%)	0.548
Infection, *n* (%)	18(7.3%)	57(22.6%)^*^	<0.001
**Laboratory results**			
ALT, U/L, IQR	19.0(13.0,29.0)	19.0(12.0,34.0)	0.823
AST, U/L, IQR	22.0(18.0,29.0)	24.0(18.0,35.3)^*^	0.042
TBIL, umol/L, IQR	16.9(11.4,25.0)	14.9(10.1,24.0)	0.116
AKP, U/L, mean(SD)	76.7 ± 25.4	84.2 ± 35.2^*^	0.007
GGT, U/L, IQR	39.0(26.0,74.0)	42.0(24.0,87.0)	0.364
TC, mmol/L, mean(SD)	4.0 ± 1.0	4.0 ± 1.2	0.557
TG, mmol/L, mean(SD)	1.3 ± 0.7	1.3 ± 1.2	0.895
FBG, mmol/L, mean(SD)	5.6 ± 1.6	5.5 ± 2.2	0.878
Cr, umol/L, mean(SD)	93.6 ± 46.4	99.0 ± 53.2	0.233
BUN, mmol/L, IQR	7.5 ± 3.8	8.1 ± 5.2	0.140
PA, mg/dL, mean(SD)	19.4 ± 6.9	18.3 ± 6.2	0.072
ALB, g/L, mean(SD)	40.0 ± 4.7	38.8 ± 4.9^*^	0.006
LDH, U/L, mean(SD)	254.7 ± 75.4	293.3 ± 169.8^*^	0.002
UA, umol/L, mean(SD)	421.3 ± 149.1	448.6 ± 154.3	0.061
WBC, × 10^9^, mean(SD)	6.6 ± 2.0	6.9 ± 2.2	0.093
NEU, %, mean(SD)	64.4 ± 9.9	66.9 ± 10.2^*^	0.008
LYM, × 10^9^, mean(SD)	1.6 ± 0.6	1.5 ± 0.6	0.093
RBC, × 10^12^, mean(SD)	4.5 ± 0.7	4.4 ± 0.8	0.087
PLT, × 10^9^, mean(SD)	211.4 ± 69.6	217.6 ± 96.9	0.428
HGB, g/L, mean(SD)	134.7 ± 22.1	130.0 ± 26.2^*^	0.035
NT-proBNP, pg/mL, IQR	3395.0 (1565.0,7063.5)	4364.0 (2269.8,9143.8)^*^	0.010
**Medication**			
ACEI/ARB, *n* (%)	187(75.4%)	196(77.8%)	0.531
β-blockers, *n* (%)	209(84.3%)	207(82.1%)	0.524
MRA, *n* (%)	216(87.1%)	221(87.7%)	0.836
Diuretics, *n* (%)	225 (90.7%)	246(97.6%)^*^	0.001
Sacubitril/Valsartan, *n* (%)	21(8.5%)	13(5.2%)	0.142
Ivabradine, *n* (%)	22(8.9%)	19(7.5%)	0.587
Aspirin, *n* (%)	166(66.9%)	146(57.9%)^*^	0.038
Statin, *n* (%)	154(62.1%)	155(61.5%)	0.892
Digoxin, *n* (%)	101(40.7%)	87(34.5%)	0.152
Cedilanid, *n* (%)	121(48.8%)	145(57.5%)	0.050
Levosimendan, *n* (%)	95(38.3%)	86(34.1%)	0.331
rhBNP, *n* (%)	40(16.1%)	80(31.7%)^*^	<0.001

* *p*-value < 0.05. SBP, Systolic pressure; DBP, Diastolic blood pressure; HR, Heart rate; HT, Hypertension; DM, Diabetes; CAD, Coronary heart disease; ECG, Electrocardiogram; ALT, Alanine aminotransferase; AST, Aspartate aminotransferase; TBIL, Total bilirubin; AKP, Alkaline phosphatase; GGT, Glutamyl transpeptidase; TC, Total cholesterol; TG, Triglyceride; Cr, Creatinine; BUN, Blood urea nitrogen; PA, Prealbumin; ALB, Albumin; LDH, Lactate dehydrogenase; UA, Uric acid; WBC, Leukocyte; NEU, Neutrophil; LYM, Lymphocyte; RBC, Red blood cells; PLT, Platelets; HGB, Hemoglobin; NT-proBNP, N-terminal-pro-B-type natriuretic peptide; ACEI, Angiotensin converting enzyme inhibitor; ARB, Angiotensin II receptor antagonist; MRA, mineralocorticoid receptor antagonism; rhBNP, recombinant human brain natriuretic peptide.

Logistic regression was performed using the hospital stays of patients with CHF as the dependent variable, and basic information, laboratory results, medicine as independent variables. The results suggested that infection, high ALT, and usage of rhBNP were risk factors for prolonging the hospital stays of patients with CHF. The usage of aspirin and Sacubitril/Valsartan were protective factors for shortening the hospital stays of patients with CHF (Table 5).

**Table 5:** Logistic regression analysis of hospital stays of patients with CHF.

	**β**	**OR**	**95%CI**	***P***
Infection	1.319	3.470	1.956–7.149	<0.001
Sacubitril/Valsartan	−1.297	0.273	0.110–0.677	0.005
Aspirin	−0.538	0.584	0.432–0.790	<0.001
rhBNP	0.771	2.163	1.340–3.488	0.002
ALT(U/L)	0.003	1.003	1.000–1.006	0.025

rhBNP, recombinant human brain natriuretic peptide; ALT, Alanine aminotransferase.

The median hospital expenses for patients with CHF were 14,894.86 Yuan. We divided all patients into the low hospital expenses group and the high hospital expenses group based on the median of hospital expenses. Compared with the low hospital expenses group, patients in the high hospital expenses group with infection showed a higher proportion (*p* < 0.05). The levels of AKP, creatinine(Cr), blood urea nitrogen(BUN), LDH, neutrophil, NT-proBNP were higher (*p* < 0.05), and Total cholesterol(TC), prealbumin, albumin, lymphocyte, red blood cell(RBC), and hemoglobin were lower (*p* < 0.05). In terms of medicine, the rates of diuretics, cedilanid, and rhBNP were higher in high hospital expenses group than in low hospital expenses group (*p* < 0.05) (Table 6).

**Table 6:** Influencing factors of hospital costs for patients with CHF.

	**Low hospital expenses group (*N* = 250)**	**High hospital expenses group (*N* = 250)**	***p***
**Basic information**			
Male sex, *n* (%)	143(57.2%)	139(55.6%)	0.718
Age, years, mean(SD)	64.7 ± 15.2	64.8 ± 14.6	0.914
SBP, mmHg, mean(SD)	124.1 ± 20.9	127.3 ± 24.3	0.117
DBP, mmHg, mean(SD)	73.8 ± 14.7	74.8 ± 15.3	0.443
HR, bpm, mean(SD)	80.7 ± 18.3	83.5 ± 21.7	0.120
HT History, *n* (%)	116(46.4%)	131(52.4%)	0.128
DM History, *n* (%)	68(27.2%)	73(29.2%)	0.619
CAD History, *n* (%)	95(38.0%)	98(39.2%)	0.927
Infection, *n* (%)	17(6.8%)	58(23.2%)^*^	<0.001
**Laboratory results**			
ALT, U/L, IQR	20.0(13.0, 28.0)	18.5(12.0, 32.3)	0.860
AST, U/L, IQR	22.0(18.0, 29.0)	24.0(18.0, 33.0)	0.058
TBIL, umol/L, IQR	15.1(10.6, 23.5)	16.2(10.7, 26.1)	0.685
AKP, U/L, mean(SD)	76.5 ± 25.2	84.4 ± 35.4^*^	0.004
GGT, U/L, IQR	38.0(27.0, 70.5)	44.0(23.0, 87.0)	0.412
TC, mmol/L, mean(SD)	4.1 ± 1.0	3.9 ± 1.2^*^	0.023
TG, mmol/L, mean(SD)	1.3 ± 0.7	1.3 ± 1.2	0.912
FBG, mmol/L, mean(SD)	5.5 ± 1.6	5.6 ± 2.3	0.446
Cr, umol/L, mean(SD)	90.9 ± 40.7	101.7 ± 57.3^*^	0.017
BUN, mmol/L, IQR	7.2 ± 3.4	8.4 ± 5.4^*^	0.005
PA, mg/dL, mean(SD)	19.9 ± 6.2	17.8 ± 6.7^*^	0.001
ALB, g/L, mean(SD)	40.2 ± 4.5	38.7 ± 5.0^*^	<0.001
LDH, U/L, mean(SD)	253.7 ± 96.9	293.6 ± 157.0^*^	0.001
UA, umol/L, mean(SD)	421.2 ± 151.0	448.6 ± 152.5	0.060
WBC, × 10^9^, mean(SD)	6.6 ± 1.9	6.9 ± 2.3	0.204
NEU, %, mean(SD)	64.0 ± 8.9	67.2 ± 10.9^*^	0.001
LYM, × 10^9^, mean(SD)	1.7 ± 0.6	1.5 ± 0.7^*^	0.003
RBC, × 10^12^, mean(SD)	4.5 ± 0.7	4.3 ± 0.8^*^	0.015
PLT, × 10^9^, mean(SD)	214.4 ± 70.9	214.8 ± 96.2	0.961
HGB, g/L, mean(SD)	134.9 ± 21.3	129.8 ± 26.8^*^	0.021
NT-proBNP, pg/mL, IQR	3168.0(1560.0, 6203.0)	4660.0(2322.5, 9760.0)^*^	<0.001
**Medication**			
ACEI/ARB, *n* (%)	191(76.4%)	192(76.8%)	0.916
β-blockers, *n* (%)	216(86.4%)	200(80.0%)	0.056
MRA, *n* (%)	221(88.4%)	216(86.4%)	0.500
Diuretics, *n* (%)	227(90.8%)	244(97.6%)^*^	0.001
Sacubitril/Valsartan, *n* (%)	22(8.8%)	12(4.8%)	0.076
Ivabradine, *n* (%)	19(7.6%)	22(8.8%)	0.625
Aspirin, *n* (%)	158(63.2%)	154(61.6%)	0.712
Statin, *n* (%)	153(61.2%)	156(62.4%)	0.782
Digoxin, *n* (%)	98(39.2%)	90(36.0%)	0.460
Cedilanid, *n* (%)	119(47.6%)	147(58.8%)^*^	0.012
Levosimendan, *n* (%)	81(32.4%)	100(40.0%)	0.077
rhBNP, *n* (%)	24(9.6%)	96(38.4%)^*^	<0.001

* *p*-value < 0.05. SBP, Systolic pressure; DBP, Diastolic blood pressure; HR, Heart rate; HT, Hypertension; DM, Diabetes; CAD, Coronary heart disease; ECG, Electrocardiogram; ALT, Alanine aminotransferase; AST, Aspartate aminotransferase; TBIL, Total bilirubin; AKP, Alkaline phosphatase; GGT, Glutamyl transpeptidase; TC, Total cholesterol; TG, Triglyceride; Cr, Creatinine; BUN, Blood urea nitrogen; PA, Prealbumin; ALB, Albumin; LDH, Lactate dehydrogenase; UA, Uric acid; WBC, Leukocyte; NEU, Neutrophil; LYM, Lymphocyte; RBC, Red blood cells; PLT, Platelets; HGB, Hemoglobin; NT-proBNP, N-terminal-pro-B-type natriuretic peptide; ACEI, Angiotensin converting enzyme inhibitor; ARB, Angiotensin II receptor antagonist; MRA, Mineralocorticoid receptor antagonism; rhBNP, Recombinant human brain natriuretic peptide.

Logistic regression was performed using the hospital expenses of patients with CHF as the dependent variable, and basic information, laboratory results, medicine as independent variables. The results suggested that infection, usage of rhBNP and high LDH were risk factors that increased the hospital expenses for patients with CHF, while the use of Sacubitril/Valsartan and high prealbumin were protective factors for reducing hospital expenses of for patients with CHF (Table 7).

**Table 7:** Logistic regression analysis of hospital expenses of patients with CHF.

	**β**	**OR**	***P***	**95%CI**
Infection	1.229	3.418	<0.001	1.731–6.747
Sacubitril/Valsartan	−1.779	0.169	0.001	0.060–0.476
rhBNP	1.803	6.069	<0.001	3.348–11.004
PA(mg/dL)	−0.040	0.960	<0.001	0.940–0.981
LDH	0.002	1.002	0.038	1.000–1.003

rhBNP, Recombinant human brain natriuretic peptide; PA, Prealbumin; LDH, Lactate dehydrogenase.

According to CONUT score, patients with CHF were divided into three groups: 143 patients were normal group, 237 patients were mild malnutrition group, 66 patients were moderate or severe malnutrition group. Compared with the normal group, patients in the mild malnutrition, moderate or severe malnutrition group had higher infection ratios, longer hospital stays, and larger hospital expenses (*p* < 0.05). Compared with the normal and mild malnutrition group, the usage ratio of ACEI/ARB, β-blocker in the moderate or malnutrition group were lower, the usage ratio of Cedilanid and rhBNP in the moderate or severe malnutrition group were higher. In terms of prognosis, the ratios of improvement in the moderate or severe malnutrition group was lower than in the normal and mild malnutrition group (*p* < 0.05). In terms of financial burden, the hospital stays and hospital expenses in the malnutrition group was higher than in the normal group (*p* < 0.05) (Table 8).

**Table 8:** Prognosis and the financial burden in patients with CHF with different nutritional status.

	**Normal group (*N =* 143)**	**Mild malnutrition group (*N =* 237)**	**Moderate or severe malnutrition group (*N =* 66)**	***p***
**Basic information**				
Male sex, *n* (%)	77(53.9%)	138(58.2%)	41(62.1%)	0.495
Age, years, mean(SD)	61.1 ± 14.5	66.0 ± 14.0^#^	68.7 ± 14.6^*^^#^	<0.001
SBP, mmHg, mean(SD)	124.2 ± 22.2	127.6 ± 23.1	125.8 ± 25.0	0.362
DBP, mmHg, mean(SD)	75.1 ± 15.3	75.3 ± 14.8	72.0 ± 14.9	0.266
HR, bpm, mean(SD)	82.6 ± 19.9	81.6 ± 20.5	81.6 ± 19.8	0.872
HT History, *n* (%)	62(43.4%)	124(52.3%)	36(54.6%)	0.168
DM History, *n* (%)	33(23.1%)	76(32.1%)	21(31.8%)	0.153
CAD History, *n* (%)	47(32.9%)	96(40.5%)	28(42.4%)	0.253
Infection, *n* (%)	10(7.00%)	41(17.3%)^#^	15(22.7%)^*^^#^	0.003
**Laboratory results**				
ALT, U/L, IQR	20.0(14.0, 30.0)	18.0(12.0, 34.5)	17.0(9.0, 28.3)	0.093
AST, U/L, IQR	22.5(19.0, 31.0)	23.0(18.0, 30.0)	23.5(18.0, 31.5)	0.930
TBIL, umol/L, IQR	13.9(10.3, 20.9)	15.8(10.9, 23.6)	19.0(8.9, 40.2)	0.117
AKP, U/L, mean(SD)	77.2 ± 24.5	78.9 ± 25.6	88.8 ± 43.6^*^^#^^+^	0.019
GGT, U/L, IQR	39.0(27.0, 67.0)	37.0(24.0, 73.0)	48.0(22.0, 87.5)	0.854
TC, mmol/L, mean(SD)	4.8 ± 0.8	3.7 ± 1.0^#^	3.3 ± 1.3^*^^#^^+^	<0.001
TG, mmol/L, mean(SD)	1.7 ± 0.9	1.2 ± 1.1^#^	1.0 ± 0.4^*^^#^	<0.001
FBG, mmol/L, mean(SD)	5.4 ± 1.6	5.6 ± 2.0	5.5 ± 2.4	0.818
Cr, umol/L, mean(SD)	88.0 ± 42.2	91.4 ± 38.0	128.6 ± 80.2^*^^#^^+^	<0.001
BUN, mmol/L, IQR	6.7 ± 3.2	7.4 ± 3.3	11.5 ± 7.8^*^^#^^+^	<0.001
PA, mg/dL, mean(SD)	22.3 ± 6.6	18.4 ± 5.7^#^	13.0 ± 4.9^*^^#^^+^	<0.001
ALB, g/L, mean(SD)	41.9 ± 3.6	39.8 ± 4.1^#^	33.9 ± 4.7^*^^#^^+^	<0.001
LDH, U/L, mean(SD)	262.4 ± 79.5	272.7 ± 117.7	278.4 ± 137.4	0.562
UA, umol/L, mean(SD)	428.7 ± 153.8	433.5 ± 152.6	463.2 ± 140.7	0.311
WBC, × 10^9^, mean(SD)	7.2 ± 2.2	6.5 ± 1.9^#^	6.3 ± 2.8^*^^#^	0.002
NEU, %, mean(SD)	61.0 ± 9.4	66.1 ± 8.9^#^	73.6 ± 9.0^*^^#^^+^	<0.001
LYM, × 10^9^, mean(SD)	2.0 ± 0.5	1.5 ± 0.5^#^	0.9 ± 0.3^*^^#^^+^	<0.001
RBC, × 10^12^, mean(SD)	4.8 ± 0.7	4.4 ± 0.6^#^	3.9 ± 0.9^*^^#^^+^	<0.001
PLT, × 10^9^, mean(SD)	241.6 ± 93.7	209.7 ± 80.0^#^	181.6 ± 73.9^*^^#^^+^	<0.001
HGB, g/L, mean(SD)	143.7 ± 22.6	131.1 ± 22.2^#^	117.7 ± 24.2^*^^#^^+^	<0.001
NT-proBNP, pg/mL, IQR	2861.5 (1333.5, 4647.5)	4153.0 (1999.0, 7379.5)	8210.5 (3548.3, 17768.3)	<0.001
**Medication**				
ACEI/ARB, *n* (%)	118(82.5%)	186(78.5%)	43(65.2%)^*^^#^	0.018
β-blockers, *n* (%)	128(89.5%)	206(86.9%)	44(66.7%)^*^^#^^+^	<0.001
MRA, *n* (%)	119(83.2%)	214(90.3%)	59(89.4%)	0.113
Diuretics, *n* (%)	131(91.6%)	224(94.5%)	65(98.5%)	0.096
Sacubitril/Valsartan, *n* (%)	11(7.7%)	18(7.6%)	1(1.5%)	0.187
Ivabradine, *n* (%)	15(10.5%)	14(5.9%)	5(7.6%)	0.265
Aspirin, *n* (%)	93(65.0%)	142(59.9%)	38(57.6%)	0.493
Statin, *n* (%)	86(60.1%)	149(62.9%)	41(62.1%)	0.868
Digoxin, *n* (%)	55(38.5%)	91(38.4%)	24(36.4%)	0.951
Cedilanid, *n* (%)	68(47.6%)	119(50.2%)	47(71.2%)^*^^#^^+^	0.004
Levosimendan, *n* (%)	53(37.1%)	90(38.0%)	20(30.3%)	0.513
rhBNP, *n* (%)	25(17.5%)	60(25.3%)	25(37.9%)^*^^#^	0.006
**Prognosis**				
Improvement	139(97.2%)	232(97.9%)	59(89.4%)^*^^+^	0.016
Multiple admissions	22(15.4%)	54(22.8%)	11(16.7%)	0.173
**Financial Burden**				
Hospital stays	10.0(8.0,13.0)	11.0(8.0,14.0)^#^	11.5(8.8,16.3)^*^^#^	0.041
Hospital costs	12971.8 (9892.7, 18499.3)	15605.2 (11650.2, 24921.8)^#^	18563.6 (14305.9, 26001.6)^*^^#^^+^	<0.001

* P-value < 0.05;

# p-value < 0.05/3 vs. Normal group;

+ p-value <0.05/3 vs. Mild malnutrition group. SBP, Systolic pressure; DBP, Diastolic blood pressure; HR, Heart rate; HT, Hypertension; DM, Diabetes; CAD, Coronary heart disease; ECG, Electrocardiogram; ALT, Alanine aminotransferase; AST, Aspartate aminotransferase; TBIL, Total bilirubin; AKP, Alkaline phosphatase; GGT, Glutamyl transpeptidase; TC, Total cholesterol; TG, Triglyceride; Cr, Creatinine; BUN, Blood urea nitrogen; PA, Prealbumin; ALB, Albumin; LDH, Lactate dehydrogenase; UA, Uric acid; WBC, Leukocyte; NEU, Neutrophil; LYM, Lymphocyte; RBC, Red blood cells; PLT, Platelets; HGB, Hemoglobin; NT-proBNP, N-terminal-pro-B-type natriuretic peptide; ACEI, Angiotensin converting enzyme inhibitor; ARB, Angiotensin II receptor antagonist; MRA, Mineralocorticoid receptor antagonism; rhBNP, Recombinant human brain natriuretic peptide.

## Discussion

Our study assessed the associations between nutrition, infection and prognosis, financial burden, and proved that COUNT was also applicable to evaluate the prognosis and financial burden of CHF inpatients in China.

“Golden Triangle” medicines-ACEI/ARB, β-blockers and MRA was called the cornerstone of CHF treatment (20). Usage ratios of ACEI/ARB and β-blockers in our study were similar to the results of prior studies (21, 22). But the usage ratio of MRA was significantly higher, which may be related to most clinicians still used MRA as potassium-preserving diuretics. Although usage ratios of medicines were similar, the ratio of improvement in our study was lower and the financial burden was heavier (21, 23). So, we need to search for other factors that may affect.

In the regression analysis, our research indicated that low albumin caused poor therapeutic outcomes, while low prealbumin increased hospital expenses and shortened the readmission interval, namely increased the frequency of hospitalizations within the same time. As a protein synthesized in the liver, albumin was an indispensable indicator for clinical evaluation of patients' nutritional status (24, 25). Prealbumin which is a precursor of albumin has a shorter half-life than albumin, and also was used to assess patients' nutritional status (26). By the ROC curve, we found that 37.45 g/L was the optimal cut-off point for albumin to predict the therapeutic outcomes of patient with CHF, which suggested that albumin supplementation was conducive to improve therapeutic outcomes for the CHF patient with albumin <37.45 g/L at admission. Previous studies had proved that inspection and intervention in a timely manner the albumin and prealbumin of inpatients was of great significance to improve prognosis and reduce hospital expenses, (27, 28) which is also applicable to Chinese inpatients with CHF.

Infection is a common cause of hospitalization in patients with CHF, especially respiratory infection (29, 30). In our study, 15% of inpatients with CHF were due to infection and respiratory infection accounts for 93.1% of the total infection. Heart failure leads to the decline of heart pumping function, pulmonary vein blood stasis in the lungs, which make patients with CHF more susceptible to infection (31). Our study found that infection increased the hospital stays and expenses of patients with CHF. In real world, hospital expenses only account for a part of out-of-pocket expenses of patients, other expenses include wages of professional caregiver, charge for loss of working time and so on (32). Therefore, we take both hospital expenses and stays as a measure to assess the financial burden of inpatients with CHF. Financial burden was an important factor influencing the CHF patient's long-term compliance, which determined final prognosis (33). Previous studies have reported the impact of infection on the prognosis and severity of heart failure (34, 35). Our study finds the importance of infection on the financial burden of inpatients with CHF. Therefore, clinicians should pay more attention to propagate the notion that avoids infection out of hospitalization in patient with CHF.

We hope finding a score enabled evaluation of the patient's protein reserve and immune defenses. CONUT compounded this requirement. It was calculated on three parameters, the serum albumin and total cholesterol levels and the total lymphocyte count. CONUT is usually used to evaluate the prognosis of cancer patients, (16, 36). Our study revealed that the ratio of infection of CHF patients with high CONUT score is higher, therapeutic outcomes is poorer, financial burden is heavier. One of the reasons for the poor prognosis of CHF patients with moderate or severe malnutrition is the low usage ratio of β-blockers. The high usage ratio of rhBNP in CHF patients with moderate or severe malnutrition increases financial burden.

RhBNP had the risk of increasing the financial burden of CHF inpatients with moderate or severe malnutrition in our study. The reason may be related to its special mode of administration. Clinically, most drugs treated HF were administered orally, but CHF inpatients with moderate or severe malnutrition were prone to acute exacerbations. For these patients, rhBNP was usually administered intravenously for 3–5 days. Naturally, used rhBNP inpatient's hospital stays was longer and the hospital expenses was higher.

Our study still had some limitations. First, there was no distinction between heart failure with preserved ejection fraction (HFpEF) and heart failure with reduced ejection fraction (HFrEF), and there were differences in therapeutic method and prognosis between HFpEF and HFrEF. However, the majority of patients undergoing cardiac ultrasound in our study were HFrEF which accounted for 69.7%. The HFpEF accounted for only 21.6%, and another 8.7% of patients were heart failure with a moderately reduced ejection fraction (HFmrEF). Second, information was collected only during the hospitalization of patients with CHF, and there was a lack of follow-up data for out-of-hospital treatment. However, during hospitalization, the patients had the most standard therapeutic schedule and the best compliance owing to supervision of doctors and nurses. It was more conducive to the analysis of influencing factors outside of medication. Third, there may be selection bias in single-center study.

## Conclusion

In conclusion, nutrition and infection significantly affected prognosis and financial burden of patients with CHF. The CONUT score, as a screening tool for assessing the nutritional and immune status of CHF patients, predicted the CHF patient's prognosis and future financial burden.

## Data availability statement

The raw data supporting the conclusions of this article will be made available by the authors, without undue reservation.

## Author contributions

LH, Z-HW, WZ, and MZ contributed to conception and design of the study. XJ organized the database. X-lY and BL performed the statistical analysis. Y-yS wrote the first draft of the manuscript. L-fS and Y-lL wrote sections of the manuscript. All authors contributed to manuscript revision, read, and approved the submitted version.

## Funding

This work was supported by the research grants from Taishan Scholars (No. tsqn202103146), Clinical Research Center of Shandong University (No. 2020SDUCRCC027), and the National Natural Science Foundation of China (82070392, 81873534, and 81800761).

## Conflict of interest

The authors declare that the research was conducted in the absence of any commercial or financial relationships that could be construed as a potential conflict of interest.

## Publisher's note

All claims expressed in this article are solely those of the authors and do not necessarily represent those of their affiliated organizations, or those of the publisher, the editors and the reviewers. Any product that may be evaluated in this article, or claim that may be made by its manufacturer, is not guaranteed or endorsed by the publisher.
