# Supplementary material for: Malnutrition and infection lead to poor prognosis and heavy financial burden of patients with chronic heart failure
Source: Front Cardiovasc Med. 2022 Dec 1;9:1045262. doi: 10.3389/fcvm.2022.1045262 (PMC9752848; doi:10.3389/fcvm.2022.1045262)
Supplement: Supplementary file 1 [file Data_Sheet_1.PDF]

Table S1

Definition of CONUT Score

Variables	Degree of undernutrition			
	Normal	Mild	Moderate	Severe
Serum albumin				
Concentration (g/dl)	≥ 3.50	3.00–3.49	2.50–2.99	< 2.50
Score	0	2	4	6
Total lymphocyte				
Count (/mm3)	≥ 1600	1200–1599	800–1199	< 800
Score	0	1	2	3
Total cholesterol				
Concentration (mg/dl)	≥ 180	140–179	100–139	< 100
Score	0	1	2	3
CONUT score = Serum albumin score + total lymphocyte score + total cholesterol score				
Total score	0–1	2–4	5–8	9–12

Table S2

Baseline Characteristics of the Study Population

	Total(N=500)
**Basic information**	
Male	282(56.4%)
Age(years)	64.8 ±14.8
BMI	24.95 ±4.15
SBP(mmHg)	125.7 ±22.7
DBP(mmHg)	74.3 ±15.0
HR(bpm)	82.1 ±20.1
HT History	247(49.4%)
DM History	141(28.2%)
CAD History	193(38.6%)
Infection	75(15.0%)

**Cardiac ultrasound**	
LVEF(%)	36.1±15.0
LAD(mm)	47.2±9.6
LVID(mm)	58.3±11.9
**Etiologies**	
Ischemic Heart Disease	268(53.6%)
Dilated Cardiomyopathy	89(17.8%)
Hypertension	61(12.2%)
Heart Valve Disease	57(11.4%)
Congenital Heart Disease	13(2.6%)
Others Etiologies	12(2.4%)
**Inducements**	
Infection	75(15.0%)
Arrhythmia	2(0.4%)
Mood Dysregulation	10(2.0%)
Others Inducements	413(82.6%)
**Auxiliary Examinations**	
ECG	500(100%)
NT-proBNP	487(97.4%)
Echocardiography	458(91.6%)
Myocardial Injury Markers	445(89.0%)
Computed Tomography	87(17.4%)
Chest Radiograph	29(5.8%)
**Medication**	
ACEI/ARB	383(76.6%)
β-blockers	416(83.2%)
MRA	437(87.4%)
Sacubitril/Valsartan	34(6.8%)
Ivabradine	41(8.2%)

Aspirin	312(62.4%)
Statin	309(61.8%)
Diuretics	471(94.2%)
Cedilanid	266(53.2%)
Digoxin	188(37.6%)
Levosimendan	181(36.2%)
rhBNP	120(24.0%)

* p value<0.05.

BMI: Body Mass Index, SBP: Systolic pressure, DBP: Diastolic blood pressure, HR: Heart rate, HT: Hypertension, DM: Diabetes, CAD: Coronary heart disease, LVEF: Left ventricular ejection fraction, LAD: Left atrial inner diameter, LVID: Left ventricular inner diameter, ECG: Electrocardiogram, NT-proBNP: N-terminal-pro-B-type natriuretic peptide, ACEI: Angiotensin converting enzyme inhibitor, ARB: Angiotensin II receptor antagonist, MRA: Mineralocorticoid receptor antagonism, rhBNP: recombinant human brain natriuretic peptide
